# A disease registry study to prospectively observe treatment patterns and outcomes in patients with HER2-positive unresectable LA/MBC: final results of the ESTHER study

**DOI:** 10.1007/s10549-025-07708-4

**Published:** 2025-05-13

**Authors:** Alistair Ring, Stephanie Sutherland, Catherine Harper-Wynne, James Owen, Thibaut Sanglier, Galina Velikova

**Affiliations:** 1 Royal Marsden Hospital NHS Foundation Trust and Institute of Cancer Research, London, UK; 2 Mount Vernon Hospital, Northwood, UK; 3 Kent Oncology Centre, Maidstone Hospital, Kent, UK; 4 Roche Products Ltd, Welwyn Garden City, UK; 5 F. Hoffmann-La Roche Ltd, Basel, Switzerland; 6 Leeds Institute of Medical Research and Leeds Teaching Hospitals NHS Trust, University of Leeds, Leeds, UK

**Keywords:** Metastatic, Breast, Cancer, HER2+, Registry, Non-interventional

## Abstract

**Purpose:**

There are multiple contemporary systemic therapy options for patients with HER2-positive advanced breast cancer. However, there are few longitudinal data regarding what proportion of patients go on to receive later lines of therapy, real-world outcomes and the impact of brain metastases. We therefore conducted a prospective, multicentre non-interventional study to describe the anti-cancer treatment regimens used and clinical outcomes in patients with HER2-positive advanced breast cancer across multiple lines of therapy undergoing treatment in routine clinical care.

**Methods:**

Adult patients diagnosed with HER2-positive advanced breast cancer were recruited to a prospective, multicentre non-interventional study to observe treatment patterns and outcomes.

**Results:**

Three hundred and eleven patients were recruited with median age 57 years. Of those patients initiating first, second-, and third-line treatment, 72 (23.2%), 59 (41.3%), and 20 (35%), respectively had passed away without advancing on to subsequent lines of therapy. The median progression-free survival in the first line was 25.8 months and overall survival 56.7 months. Over the course of the study 107 (34.4%) of participants were diagnosed with CNS metastases. Median overall survival from diagnosis of brain metastases was 15.4 months.

**Conclusions:**

Many patients treated in routine practice may not get to benefit from contemporary second and later line treatments, where brain metastases become increasingly common. These findings have implications for selection of optimal systemic therapy sequencing in advanced HER2-positive breast cancer.

**Clinical Trial Registration:**

This study was approved by Nottingham Research Ethics Committee on 29th December 2014. Clinical Trial Registration: NCT02393924.

**Supplementary Information:**

The online version contains supplementary material available at 10.1007/s10549-025-07708-4.

## Background

Over-expression or amplification of the human epidermal growth factor receptor 2 (HER2), which is present in 15–30% of breast cancers, is associated with a more aggressive clinical phenotype and a worse prognosis [1, 2]. However, the introduction of anti-HER2-targeted therapies, including monoclonal antibodies, antibody–drug conjugates and small molecule tyrosine-kinase inhibitors has considerably increased the treatment options for such patients [3].

These therapies have shown efficacy in clinical trials which have recruited patients in restricted populations, which often focus on a single line of therapy defined by prior lines of therapy and previous treatments received. The populations recruited to the registration trials may not be representative of the population of patients seen in clinic. In particular, historically patients with brain metastases were either completely excluded from trials or often under-represented by exclusion criteria related to stability of CNS disease or steroid use. Furthermore, these trials do not examine outcomes across the whole treatment pathway involving subsequent lines of therapy. Therefore, despite all of the available clinical trial data, relatively little is known about outcomes in routine care, including what proportion of patients are likely to proceed to later lines of therapy, toxicity and the cumulative rate of brain metastases.

We therefore conducted a prospective, multicentre non-interventional study to describe the anti-cancer treatment regimens used and clinical outcomes in patients with HER2-positive advanced breast cancer across multiple lines of therapy undergoing treatment in routine clinical care.

The primary objective for this study was to observe the different anti-cancer treatment regimens and their sequencing throughout the course of the disease and to describe clinical outcome for each anti-cancer treatment regimen measured as progression-free survival (PFS). Secondary objectives aimed to describe the characteristics of patients receiving first and subsequent line treatment, to observe overall survival (OS), clinical benefit rate (CBR) and cumulative rates of central nervous system (CNS) metastases. We also evaluated the safety profiles of different anti-cancer treatment regimens through the reporting of serious adverse events (SAEs), specific adverse events relevant to HER2-targeted therapies (including cardiac toxicity) and AEs leading to treatment discontinuation or dose modification of an anti-cancer therapy.

## Methods

Eligible patients were males or females aged 18 years or older diagnosed with HER2-positive unresectable locally advanced or metastatic breast cancer (advanced breast cancer) within the last 6 months prior to enrolment. At each centre, all eligible subjects were invited to participate in the study and enrolled sequentially. No other pre-selection criteria were applied. Written informed consent was obtained and enrolled subjects received treatment and clinical assessments as determined by their treating physician, according to the standard of care and routine clinical practice at each site.

Treatment regimens were defined as any anti-cancer medication, used as a single agent or as part of a combination of medications, given from the date of initiation until the date of disease progression. Treatment regimens were grouped in 13 mutually exclusive groups according to the therapeutic class present in the regimen (e.g., ‘Pertuzumab, trastuzumab and chemotherapy’ for pertuzumab, trastuzumab and docetaxel) (Supplementary Table 1).

Follow-up visits were determined by the treating physician, and source data were collected approximately every 3 months from subject charts, clinical notes, and diagnostic and laboratory test results. All anti-cancer treatment changes, clinical outcomes (including disease progression), adverse events and survival status were collected. Subjects were considered on study until death, withdrawal of consent, loss to follow-up, or end-of-study, whichever came first.

This study had a planned sample size of approximately 300 patients. When study sizes of 100 to 1000 were examined, increasingly higher sample sizes up to 300 patients resulted in noticeably increased precision (tighter 95% CI) in measuring PFS of up to 10 months. Increasing the sample size beyond 300 did not yield a substantial increase in precision.

Descriptive statistics were used to evaluate the effectiveness and safety of treatment regimen in all enrolled participants. All tests performed were two-sided with a 5% alpha error rate, without correction for multiple comparisons. The analysis of progression-free survival (PFS) and overall survival (OS) was based on the survivor function, which represents the probability of remaining event-free from the start date of the first systemic treatment date to the first event of interest (e.g., progression or death) or the last visit. The survival function was estimated using the Kaplan–Meier method and summarized by presenting the range, 25 th and 75 th percentiles, the median survival, and a 95% confidence interval for the median. The cumulative incidence function was used to describe the incidence of brain metastases among those who were free of brain metastases at the time the first antineoplastic treatment was initiated. The proportion of participants experiencing at least one serious adverse event during the follow-up was estimated with 95% Clopper-Pearson confidence intervals (CIs).

## Results

Between February 2015 and April 2018, 311 eligible patients were recruited from 29 UK cancer centres. The end of the study occurred 5 years after the last subject was enrolled, with the last patient, last visit occurring on 19th April 2023.

Of the 311 eligible patients, 85 (27.3%) presented with de novo advanced breast cancer and 226 (72.7%) had relapsed following treatment for previous early breast cancer. The baseline characteristics of the study population are shown in Table 1. Two hundred and eight (66.9%) of the patients had hormone receptor (HR) positive (and HER2-positive) breast cancer and the mean number of metastatic sites was 2.2 (SD 1.23). In those 226 patients who had relapsed, HER2 status was confirmed in metastatic tissue only in 63 (27.9%) patients, early tissue only in 136 (60.2%) patients, and both early and metastatic tissue in 27 (11.9%) patients.

**Table 1:** Baseline patient and tumour characteristics, number (%), *n* = 311

	De novo (*n* = 85)	Relapsed (*n* = 226)	Total (*n* = 311)
Gender [n (%)]			
Female	85 (100.0)	225 (99.6)	310 (99.7)
Male	0	1 (0.4)	1 (0.3)
Age (years)			
Median	55.0	58.0	57.0
Range	24–96	28–89	24–96
LVEF (%) at baseline			
Median	66.0	62.0	62.0
Range	36–85	50–81	36–85
ECOG [n (%)]*			
0–1	47 (55.3)	113 (10.3)	160 (51.4)
≥ 2	7 (8.2)	13 (4.5)	20 (6.4)
Unknown	31 (36.5)	100 (44.2)	131 (42.1)
ER/PgR [n(%)]*			
Positive	51 (60)	157 (69.5)	208 (66.9)
Negative	29 (34.1)	59 (26.1)	88 (28.3)
Unknown	5 (5.9)	10 (4.4)	15 (4.8)
Sites of metastasis (%)			
Bone	42 (49.4)	103 (45.6)	145 (46.6)
Liver	40 (47.1)	77 (34.1)	117 (37.6)
Lung	24 (28.2)	76 (33.6)	100 (32.2)
Pleural Effusion, Chest Wall** & Mediastinum	4 (4.8)	42 (18.6)	46 (14.9)
Brain/CNS	2 (2.4)	19 (8.4)	21 (6.8)
Abdominal Non-Hepatic & Ascites	1 (1.2)	8 (3.6)	9 (2.9)
Other	6 (7.1)	26 (11.5)	32 (10.3)
Number of metastatic sites (mean, SD)*	2.4 (1.36)	2.2 (1.18)	
Disease Status [n (%)]			
Locally Advanced	–	–	24 (7.7)
Metastatic	–	–	287 (92.3)
Time between initial and advanced diagnosis (months)			
Relapse within 6 months	–	6 (2.7)	–
Relapse 6–12 months	–	6 (2.7)	–
Relapse > 12 months	–	214 (94.6)	–
Pre-Study Anti-Cancer Treatments for early breast cancer			
Chemotherapy	–	165 (73.0)	–
Neoadjuvant [n (%)]	–	59 (26.1)	–
Adjuvant [n (%)]	–	114 (50.4)	–
Targeted Therapy		126 (55.8)	–
Neoadjuvant [n (%)]	–	31 (13.7)	–
Trastuzumab [n (%)]	–	30 (96.8)	–
Pertuzumab [n (%)]	–	4 (12.9)	–
Adjuvant [n (%)]	–	105 (46.5)	–
Trastuzumab [n (%)]	–	105 (100)	–
Pertuzumab [n (%)]	–	2 (1.9)	–
Hormonal Therapy	–	93 (41.2)	–
Neoadjuvant [n (%)]	–	6 (2.7)	–
Adjuvant [n (%)]	–	87 (38.5)	–
Radiotherapy related to early stage breast cancer [n (%)]			
Yes	–	156 (69.0)	–
No	–	70 (31.0)	–
Surgery related to early stage breast cancer [n (%)]			
Yes	–	206 (91.2)	–
No	–	20 (8.8)	–

^*^Percentage calculated based on patients with available data

^**^Includes skin, subcutaneous tissue and/or rib(s)

**Fig. 1:**
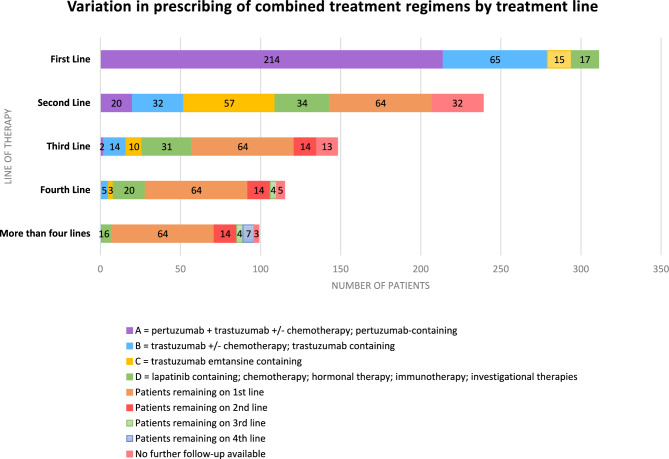
Treatments received according to treatment line, as of final data-lock (5 years after last subject enrolled), *n* = 311, entering first line treatment

In total, 125 different treatment regimens were documented across all treatment lines in this study. In the first line, 214 (68.8%) patients received pertuzumab-based regimens, 65 (20.9%) patients received trastuzumab-based regimens and 15 (4.8%) patients received trastuzumab-emtansine-based regimens. In total, 111 (35.7%) patients received hormonal therapy in first line, with 13 (4.2%) patients receiving hormonal therapy only (Fig. 1).

The disposition of patients as of data-cut-off is shown in Supplementary Fig. 1. One hundred and forty-three patients (45.9%) had progressed on to second line treatment, 64 (20.6%) remained on first line treatment, 72 (23.2%) were deceased before moving on to second line treatment, with further follow-up not available in the remaining 32 (10.2%).

In the second line, 57 patients received a trastuzumab-emtansine-based regimen (39.9%); 32 patients received a trastuzumab-based regimen (22.4%); 20 patients received a pertuzumab-based regimen (14.0%); 34 patients received other combinations (23.7%).

Treatments received in all lines are shown in Fig. 1. A notable proportion of patients initiating first, second, and third-line treatment had passed away without advancing on to subsequent lines of therapy: 72 (23.2%), 59 (41.3%), and 20 (35%), respectively. It is also likely that additional patients who were still undergoing the previous line of treatment at the time of this analysis and for whom follow-up data were unavailable, may similarly have died before advancing on to subsequent lines.

**Fig. 2:**
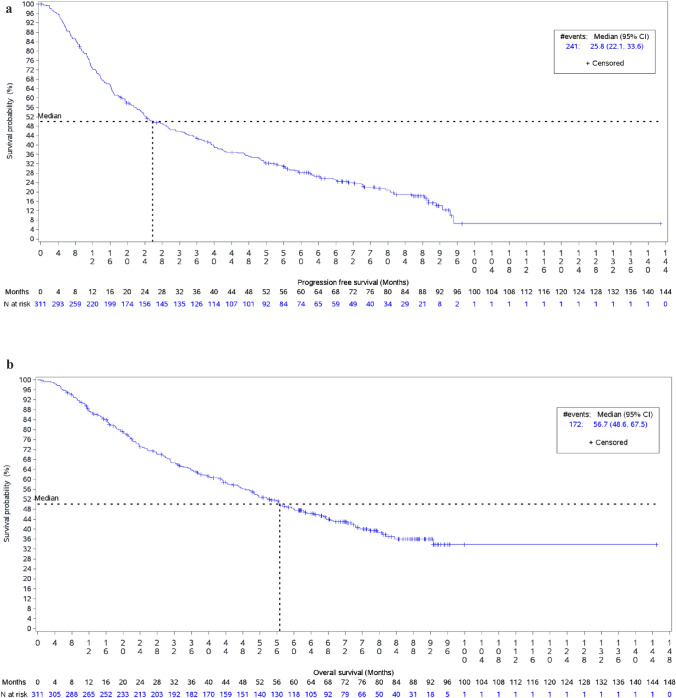
a. Progression-free survival in all first-line patients (*n* = 311). **b.** Overall survival in all first line patients (n = 311)

The median progression-free survival time during first line of treatment was 25.8 months (95% CI 21.1, 33.6), Fig. 2a. The median PFS time in the first line in those receiving pertuzumab, trastuzumab and chemotherapy was 35.9 months (95% CI 25.8, 42.0), in those receiving trastuzumab and chemotherapy was 18.6 months (95% CI 11.7, 33.4) and in patients treated with trastuzumab-emtansine was 6.9 months (95% CI 4.1, 16.2).

One hundred and ten (35.4%) patients were aged 65 or older at time of diagnosis of advanced breast cancer. The median progression-free survival time in those aged 65 years or older (23.3 months (95% CI 16.4, 39.2)) was similar to that observed in patients aged less than 65 (27.6 months (95% CI 22.1, 35.8)).

The median overall survival time was 56.7 months (95% CI 48.6, 67.5). Figure 2b. The median OS in those receiving pertuzumab, trastuzumab and chemotherapy as a first line treatment was 67.0 months (95% CI 51.8, NR), in those receiving trastuzumab and chemotherapy was 56.4 months (95% CI 33.1, 77.8) and in patients treated with trastuzumab-emtansine was 16.2 months (95% CI 6.9, 24.4).

Clinical benefit rates (complete or partial response, or stability for at least 180 days as defined by local investigator) were 70.4%, 34.3%, 28.1%, and 9% in the first to fourth lines of treatment, respectively.

**Fig. 3:**
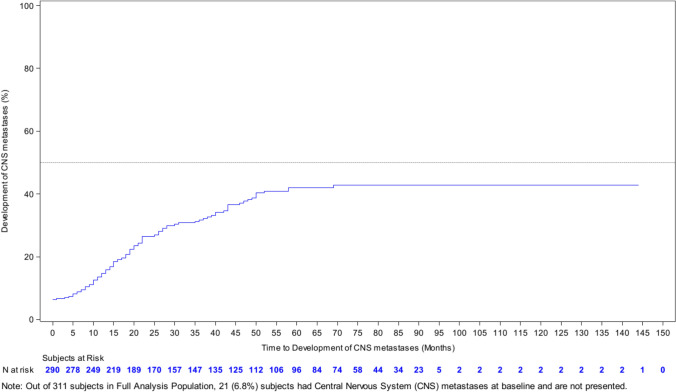
Time to development of central nervous system (CNS) metastases

At the time of diagnosis of advanced disease (study entry) 21 (6.8%) of patients were known to have CNS metastases, with a further 59 (19.0%) developing CNS metastases during first line. Of the patients entering second line treatment 33/143 (23.1%) had CNS metastases, third line 20/57 (35.1%) and fourth line 9/28 (32.1%). Over the course of the study a total of 107 (34.4%) of the study population were diagnosed with CNS metastases. Of these 107 patients, 61 (57%) had HR-positive disease, 40 (37.3%) had HR-negative disease (6 patients (5.6%) unknown HR status). Additionally, 83 patients (77.6%) were aged 18–64, 23 (21.5%) patients were aged 65–84 and 1 (0.9%) patients was aged 85 or over. The cumulative rate of development of brain metastases is shown in Fig. 3. Fifty (46.7%) of these patients had whole brain radiotherapy, 25 (23.4%) stereotactic radiotherapy, 15 (14%) had surgery alone and 17 (15.9%) had surgery with subsequent radiotherapy. The median overall survival of the patients with CNS metastases was 15.4 months (95% CI, 10.6, 25.0) (calculated from the time of development of CNS metastases) Supplementary Fig. 2.

Overall, 49 (15.8%) of patients reported adverse events leading to dose modification during first line treatment. The most common adverse events leading to dose modification were gastrointestinal toxicity 17 (5.5%) and neurological toxicity 17 (5.5%). Fifty-six (18%) of patients discontinued first line treatment due to adverse effects. The adverse events leading to discontinuation of first line treatment are listed in Supplementary Table 2.

**Fig. 4:**
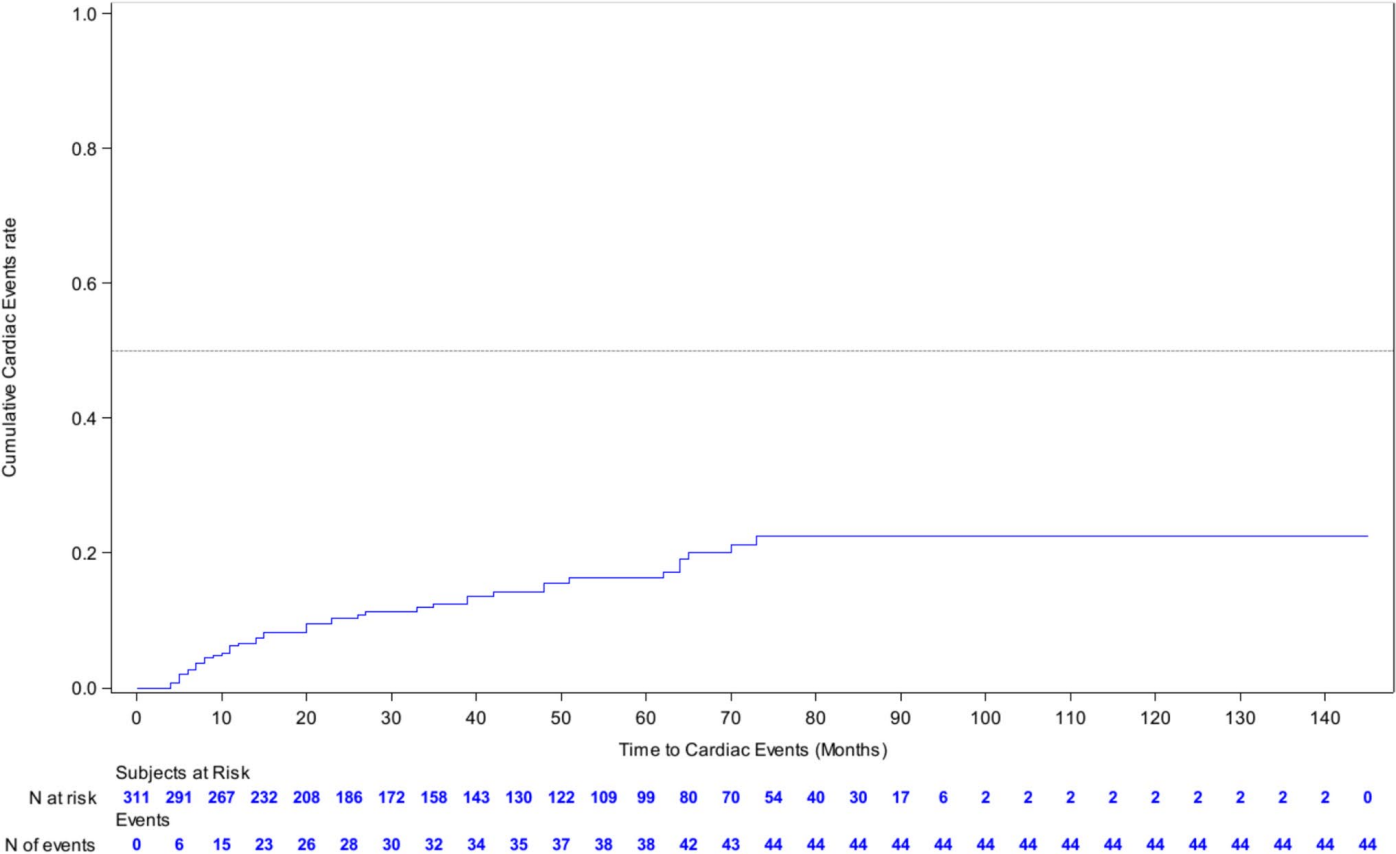
Cumulative rate of cardiac toxicity (defined as: cardiac arrest, cardiac ischaemia/infarction, congestive cardiac failure and drop in left ventricular ejection fraction to 50% or lower [and/or considered clinically significant])

Cardiac dysfunction events were defined as cardiac arrest, cardiac ischaemia/infarction, congestive cardiac failure and drop in left ventricular ejection fraction to 50% or lower (and/or considered clinically significant). Cardiac monitoring was performed as per local investigator protocol; the mean time between left ventricular assessments was 4.0 months before 19 months of treatment, and 12.3 months after the first 19 months of treatment. Overall, 44 (14.1%) of patients experienced at least one cardiac dysfunction event. Cardiac risk increased over time, and the estimated cumulative incidence rate of cardiac events at 72 months was 22.6%. Figure 4.

## Discussion

The ESTHER study describes a real-world population of patients treated within the UK, enrolled in an observational study, between 2015 and 2018, and followed up for a minimum of 5 years. The baseline characteristics were similar to patients enrolled in the ESME study: a nationwide observational cohort which gathered data of all consecutive patients with metastatic breast cancer (MBC) who initiated their treatment in 18 French Cancer Centres between 2008 and 2017 [4]. In contrast, the SystHERs study [5], a prospective registry of 977 patients in the US with HER2-positive MBC (recruited between 2012 and 2016) reported a higher proportion of patients presenting with de novo metastatic breast cancer (49.8% compared with 27% in ESTHER).

The median progression-free survival of patients receiving pertuzumab/trastuzumab and taxanes was superior to that observed in the CLEOPATRA and PERUSE studies (35.9 months, as compared to 18.5 and 20.7 months, respectively) [6, 7]. However, it is important to note that less frequent response assessment in routine practice together with less rigorous response assessment (RECIST measurements will not have been routinely conducted) will have biased in favour of a longer median PFS time. It is also important not to make comparisons between different first line therapies, as taxane/pertuzumab/trastuzumab will have been reserved for the fitter patients with de novo HER2-positive breast cancer (or at least 12 months from completion of adjuvant chemotherapy/anti-HER2 therapy) and first line trastuzumab-emtansine for those relapsing within 6 months of adjuvant systemic therapy, representing very different populations of patients.

The overall survival of patients in ESTHER who received first line pertuzumab/trastuzumab combinations was 67 months, which compared favourably with the first line CLEOPATRA and PERUSE studies (56.5 and 65.3 months, respectively) [6, 7]. The median overall survival in the whole population was 56.7 months: the SystHERs reported similar median OS of 53 months in hormone receptor positive patients and 43.4 months in hormone receptor negative patients [5]. The ESME study reported incremental improvements in median overall survival amongst patients with HER2-positive MBC (2008: 39.1 months (95% CI 36.2–46.5); 2013: 58 months (95% CI 52.0–68.4); not reached from 2014 onwards) [4]. Taken together these data suggest that the improvements in outcomes seen in first line treatment in clinical trials are reflected in clinical practice.

Since the publication of the original first line pertuzumab studies, new therapies have been developed for the treatment of advanced HER2-positive breast cancer [3]. During the time period when the ESTHER study recruited multiple lines of treatment were available for the treatment of HER2-positive advanced breast cancer, yet it is clear from the data presented that many patients die before being able to receive another line of treatment, with a maximum of 76.8% receiving 2nd line treatment and 47.6% receiving third line treatment. These data are consistent with historical data from the US [8], an analysis of a French reimbursement database [9] and an analysis of the US Flatiron database [10]. The latter study demonstrated that of 1390 patients with a documented second line of therapy for HER2-positive MBC, 34.6% had two lines only, 25.8% had three and 39.6% had four or more lines. The fact that a significant number of patients do not proceed to subsequent therapy lines is an important observation. This may reflect the fact that these were real-world patients with likely lower performance scores, more visceral compromise and more co-morbidities than would be expected from the experience in clinical trial populations.

These data underline the importance of close clinical and radiological monitoring for progression, and the importance introducing the best available treatments early, rather than relying on less effective (but potentially less toxic) intervening lines of treatment on the assumption that more effective treatments will be used at progression.

One reason that patients may clinically deteriorate and not receive multiple lines of systemic therapy is the emergence of brain metastases, which are a well-documented feature of HER2-positive breast cancer [11]. In the present study, 6.8% of patients had brain metastases at the time of diagnosis of advanced disease, with an additional 27.7% developing brain metastases over the course of the study. These results align with a large real-world dataset from the US [12]. Baseline brain imaging was not required for study entry and most UK centres do not routinely screen for brain metastases in asymptomatic patients with HER2-positive advanced breast cancer. These metastases are likely to have presented clinically. In 2022, the American Society of Clinical Oncology produced updated guidelines on the management of brain metastases in patients with HER2-positive breast cancer and came to the conclusion that there were insufficient data to recommend for or against routine magnetic resonance imaging to screen for brain metastases [13]. However, over the last few years a number of systemic therapies with activity against brain metastases have been identified, including tucatinib, an oral selective inhibitor of the HER2 tyrosine-kinase [14], and the antibody–drug conjugate Trastuzumab-Deruxtecan [15]. The availability of these therapies, and the fact that symptomatic brain metastases may compromise fitness for future therapy lines, may suggest that screening for brain metastases should be re-visited.

No new or unexpected toxicities were found in this analysis. However, one important finding was that new episodes of cardiac toxicity did continue to occur during the follow-up period. This suggest that monitoring for cardiac toxicity should continue, even in patients who have been on anti-HER2 therapy for a number of years.

This study has a number of limitations. Sites were asked to recruit patients sequentially, but it is possible that less fit patients were not recruited as they were not suitable for approach about a clinical study. Furthermore, and as discussed above, as patients were managed as standard of care, tumour assessments (for response rate and progression-free survival) were not conducted at mandated intervals.

Overall, this study demonstrates that the median overall survival in patients diagnosed with advanced HER2-positive breast cancer, treated in routine practice, now approaches 5 years. This is consistent with the published clinical trial data. Despite the widespread availability of multiple lines of therapy for this patient population, many patients receive a relatively limited number of lines of treatment and may not get to benefit from contemporary second and third line treatments. Careful consideration should therefore be taken when making decisions regarding optimal sequencing of treatment, and clinicians and patients should exercise vigilance for clinical deterioration including the development of brain metastases.

## Supplementary Information

Below is the link to the electronic supplementary material.

Supplementary file1 (DOCX 604 KB)

## Data Availability

Data supporting the results of this study can be requested via the following link: https://www.roche.com/innovation/process/clinical-trials/data-sharing/request.
