# Supplementary material for: A disease registry study to prospectively observe treatment patterns and outcomes in patients with HER2-positive unresectable LA/MBC: final results of the ESTHER study
Source: Breast Cancer Res Treat. 2025 May 13;212(1):113–21. doi: 10.1007/s10549-025-07708-4 (PMC12086108; doi:10.1007/s10549-025-07708-4)
Supplement: Supplementary file 1 — Supplementary file1 (DOCX 604 KB) [file 10549_2025_7708_MOESM1_ESM.docx]

**Appendix**

**Supplementary Figure 1. Subject disposition by treatment Line**


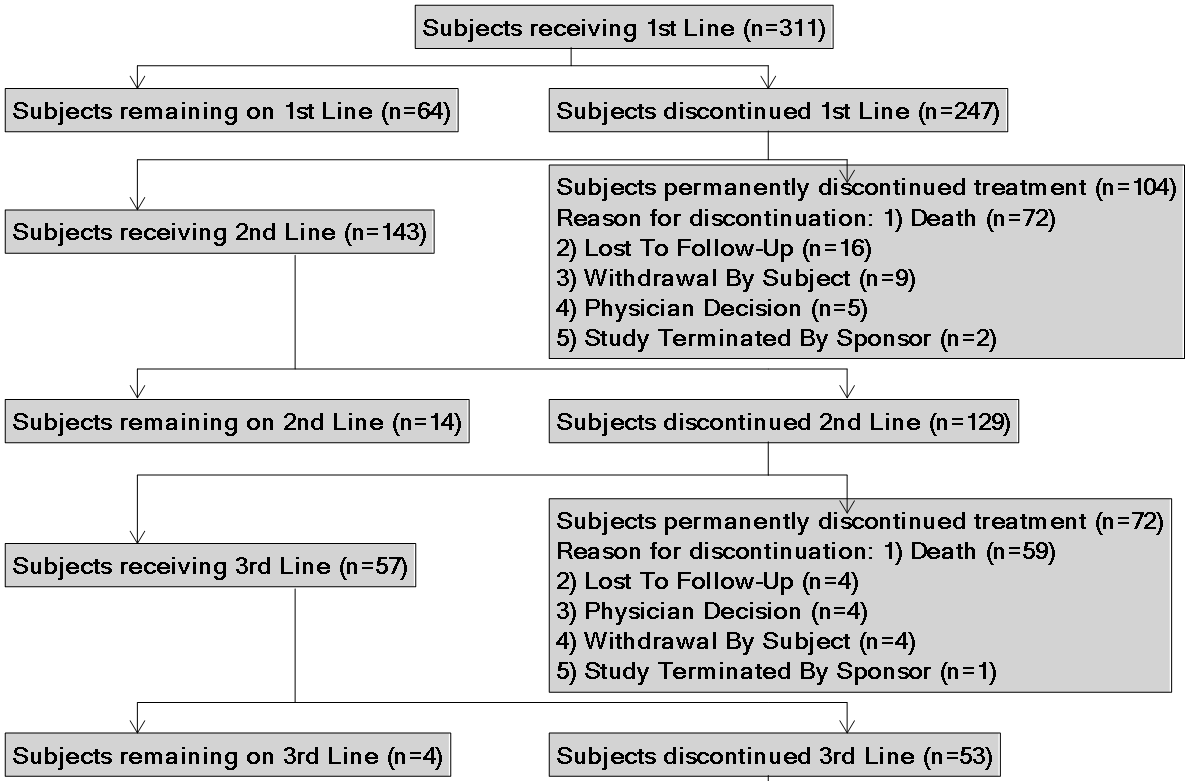

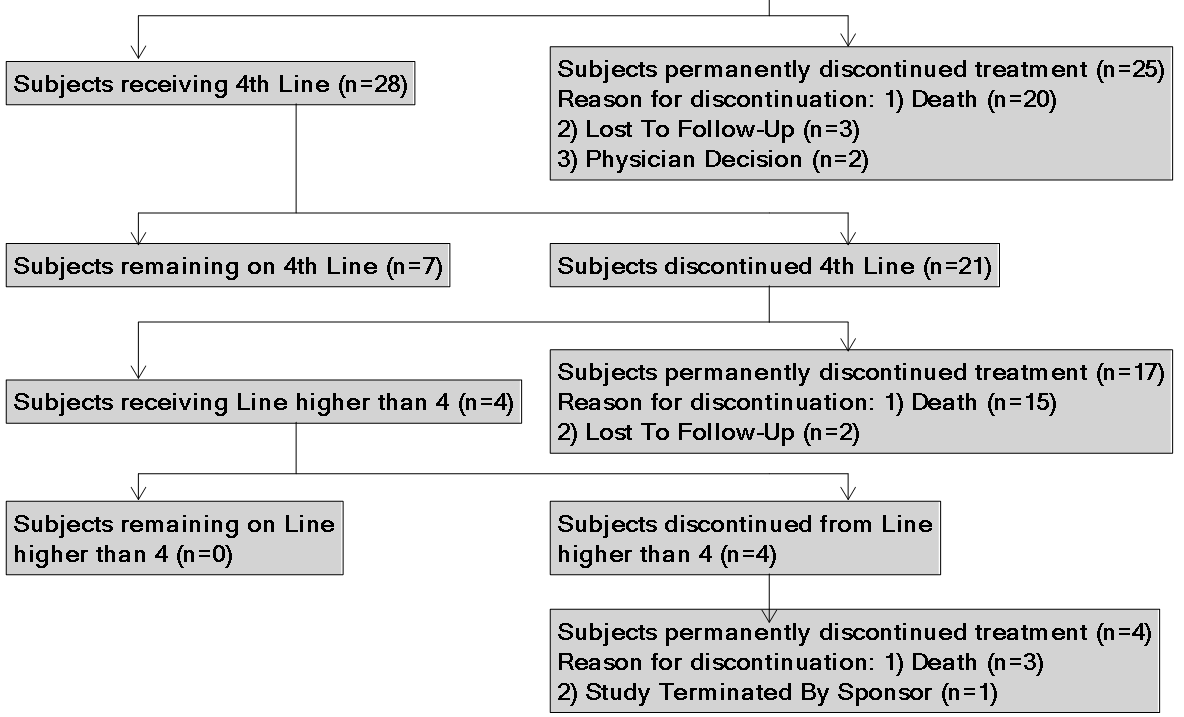


**
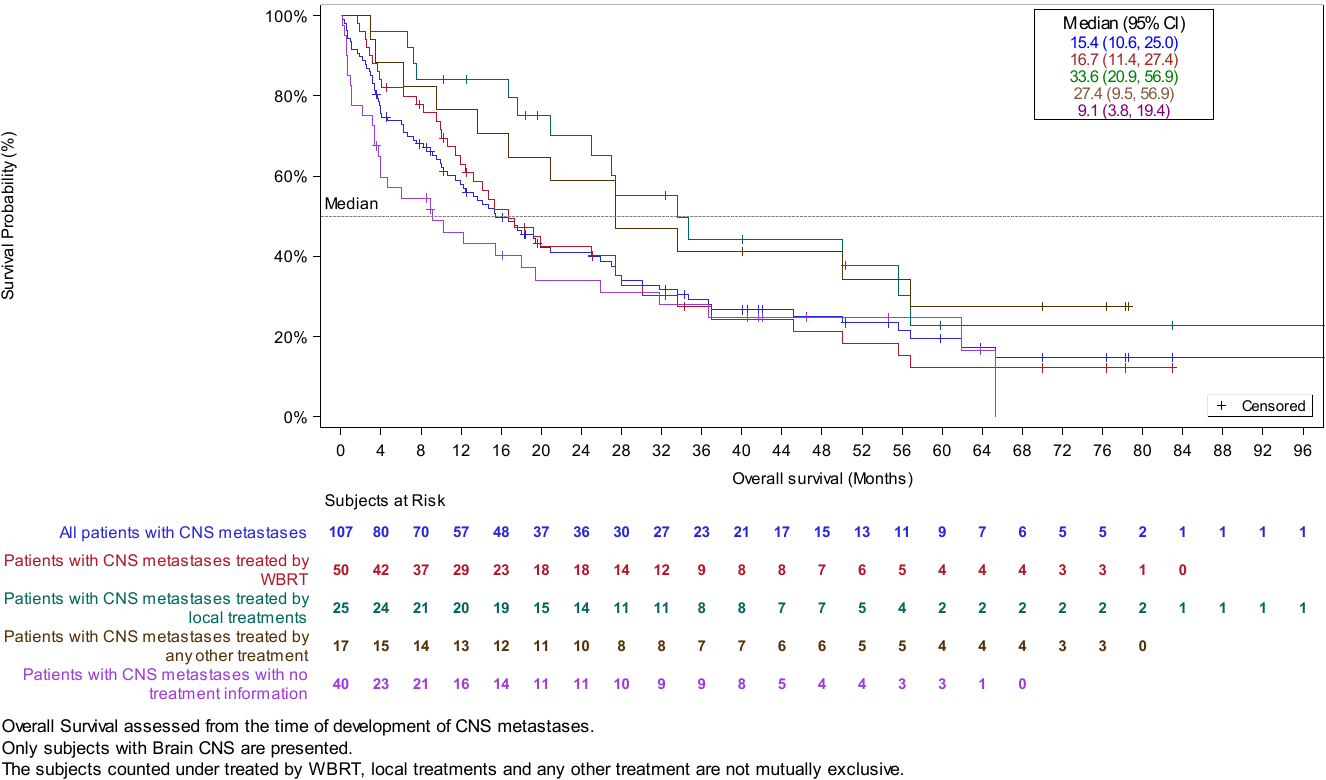
Supplementary Figure 2. Overall survival in patients with CNS metastases by treatment type.**

**Supplementary Table 1. Treatment regimen pooling**

|  | **Treatment Regimen Display** | **Description** |
| --- | --- | --- |
| 1 | Pertuzumab, trastuzumab and chemotherapy | Any combination of *pertuzumab, trastuzumab* and *any chemotherapy* |
| 2 | Pertuzumab and trastuzumab | Any combination of *pertuzumab* and *trastuzumab* without *any chemotherapy* |
| 3 | Pertuzumab-containing | Any other combination of treatment containing *pertuzumab* |
| 4 | Trastuzumab and chemotherapy | Any combination of *trastuzumab* and *any chemotherapy* |
| 5 | Trastuzumab-containing | Any treatment regimen containing *trastuzumab* not listed previously |
| 6 | Trastuzumab-emtansine | Any combination of treatment regimen containing *trastuzumab-emtansine* |
| 7 | Lapatinib-containing | Any treatment regimen containing *lapatinib* not listed previously |
| 8 | Other anti-HER2 and chemotherapy | Any combination of treatment containing *trastuzumab*, *pertuzumab*, *trastuzumab-emtansine*, *lapatinib* and/or *investigational HER2 targeted therapy* with any chemotherapy |
| 9 | Other antiHER-2 | Any combination of treatment containing *trastuzumab*, *pertuzumab*, *trastuzumab-emtansine*, *lapatinib* and/or i*nvestigational HER2 targeted therapy, without chemotherapy,* not listed previously |
| 10 | Chemotherapy-containing | All treatments within the treatment regimen are chemotherapies |
| 11 | Hormonal therapy only | All treatments within the treatment regimen are hormonal therapies |
| 12 | Immunotherapy-containing | Any treatment regimen including at least one immunotherapy not already included in the above categories |
| 13 | No Systemic Anti-Cancer Therapy | No anti-cancer treatment given (subject may have received radiotherapy or undergone surgeries) |

**Summary of discontinuations of the study due to adverse events**

- Overall **13 ( 4.2%)** **patients** discontinued the study due to adverse events on **first-line treatment**
  - **Death**
    - 1 patient in the  **pertuzumab + trastuzumab + chemotherapy**group
    - 1 patient in the  **trastuzumab + chemotherapy**group
    - 1 patient in the **hormonal therapy only** group
  - **Pneumonia**
    - 1 patient in the **trastuzumab + chemotherapy** group
    - 1 patient in the **trastuzumab-containing** group
    - 1 patient in the **trastuzumab emtansine-containing** group
  - **Pneumonitis/Respiratory failure**
    - 2 patients in the **pertuzumab + trastuzumab + chemotherapy**group
  - **Cardiac arrest**
    - 1 patient in the**pertuzumab + trastuzumab + chemotherapy** group
  - **Groin pain/Pain in extremity**
    - 1 patient with 2 events in the **pertuzumab + trastuzumab + chemotherapy**group
  - **Gastrointestinal carcinoma**
    - 1 patient in the **pertuzumab + trastuzumab + chemotherapy** group
  - **Completed suicide**
    - 1 patient in the **pertuzumab + trastuzumab** group
  - **Aortic dissection**
    - 1 patient in the **pertuzumab + trastuzumab + chemotherapy** group
- In addition, **9 ( 6.3%)** **patients** discontinued due to an adverse event on **second-line therapy**
  - 1 case of **Pneumonia**,  1 case of **Lower respiratory tract infection,  1** case of **death,**

1 case of **Cardiac** **dysfunction,** 1 case of  **Rectal** **haemorrhage**in the **trastuzumab emtansine-containing** group

- - 1 case of **Hepatic** **fibrosis**in the **trastuzumab-containing**group
  - 1 case of **Pneumonia** in the **pertuzumab-containing**group
  - 1 case of **Pneumonia**in the **chemotherapy-containing**group
  - 1 case of **death**in the **hormonal therapy only**
- In addition, **1 ( 1.8%)** **patient** discontinued due to an adverse event on **third-line therapy**
  - 1 case of **death** in the **trastuzumab containing** group

**Supplementary Table 2. Adverse events leading to discontinuation of anti-cancer medication in first line of treatment (1/6)**

- **56 (18.0%)**patients reported AEs leading to discontinuation on **first-line treatment**

| **System organ class**  **Preferred term (reported in >1  patient)**  **n (%)** | **Pertuzumab +   trastuzumab +  chemotherapy**  **(n=211)** | **Trastuzumab +**  **chemotherapy**  **(n=45)** | **Trastuzumab emtansine-  containing**  **(n=15)** |
| --- | --- | --- | --- |
| **Gastrointestinal disorders** | **9 ( 4.3)** | **3 ( 6.7)** | **0** |
| Diarrhoea | 7 ( 3.3) | 0 | 0 |
| Nausea | 0 | 2 ( 4.4) | 0 |
| Abdominal distention | 1 ( 0.5) | 0 | 0 |
| Colitis ischaemic | 1 ( 0.5) | 0 | 0 |
| Gastric ulcer perforation | 1 ( 0.5) | 0 | 0 |
| Oral disorder | 0 | 1 ( 2.2) | 0 |
| Vomiting | 1 (0.5) | 0 | 0 |

**Adverse events leading to discontinuation of anti-cancer medication in first line of treatment (2/6)**

| **System organ class**  **Preferred term (reported in >1  patient)**  **n (%)** | **Pertuzumab +   trastuzumab +  chemotherapy**  **(n=211)** | **Trastuzumab +**  **chemotherapy**  **(n=45)** | **Trastuzumab emtansine-  containing**  **(n=15)** |
| --- | --- | --- | --- |
| **General disorders and administration site conditions** | **7 ( 3.3)** | **5 (11.1)** | **0** |
| Fatigue | 4 ( 1.9) | 3 ( 6.7) | 0 |
| Chest discomfort | 1 (0.5) | 0 | 0 |
| Chest pain | 1 (0.5) | 0 | 0 |
| Death | 0 | 1 ( 2.2) | 0 |
| Oedema peripheral | 0 | 1 ( 2.2) | 0 |
| Pain | 1 (0.5) | 0 | 0 |
| Peripheral swelling | 1 (0.5) | 0 | 0 |

**Adverse events leading to discontinuation of anti-cancer medication in first line of treatment (3/6)**

| **System organ class**  **Preferred term (reported in >1  patient)**  **n (%)** | **Pertuzumab +   trastuzumab +  chemotherapy**  **(n=211)** | **Trastuzumab +**  **chemotherapy**  **(n=45)** | **Trastuzumab emtansine-  containing**  **(n=15)** |
| --- | --- | --- | --- |
| **Nervous system disorders** | **9 ( 4.3)** | **2 ( 4.4)** | **1 ( 6.7)** |
| Neuropathy peripheral | 4 ( 1.9) | 2 ( 4.4) | 0 |
| Dizziness | 1 ( 0.5) | 0 | 0 |
| Headache | 1 ( 0.5) | 0 | 0 |
| Lethargy | 1 ( 0.5) | 0 | 0 |
| Paraesthesia | 1 ( 0.5) | 0 | 0 |
| Peripheral sensory neuropathy | 1 ( 0.5) | 0 | 0 |
| Presyncope | 1 ( 0.5) | 0 | 0 |
| Seizure | 0 | 0 | 1 ( 6.7) |
| **Investigations** | **7 ( 3.3)** | **2 ( 4.4)** | **0** |
| Ejection fraction decreased | 6 ( 2.8) | 1 ( 2.2) | 0 |
| Alanine aminotransferase increased | 0 | 1 ( 2.2) | 0 |
| Weight decreased | 1 ( 0.5) | 0 | 0 |

**Adverse events leading to discontinuation of anti-cancer medication in first line of treatment (4/6)**

| **System organ class**  **Preferred term (reported in >1  patient)**  **n (%)** | **Pertuzumab +   trastuzumab +  chemotherapy**  **(n=211)** | **Trastuzumab +**  **chemotherapy**  **(n=45)** | **Trastuzumab emtansine-  containing**  **(n=15)** |
| --- | --- | --- | --- |
| **Respiratory, thoracic and  mediastinal disorders** | **6 ( 2.8)** | **1 ( 2.2)** | **0** |
| Pulmonary embolism | 1 ( 0.5) | 1 ( 2.2) | 0 |
| Dyspnoea | 2 ( 0.9) | 0 | 0 |
| Pneumonitis | 1 ( 0.5) | 1 ( 2.2) | 0 |
| Interstitial lung disease | 1 ( 0.5) | 0 | 0 |
| Respiratory failure | 1 ( 0.5) | 0 | 0 |
| **Infections and infestations** | **5 ( 2.4)** | **0** | **1 ( 6.7)** |
| Pneumonia | 1 ( 0.5) | 0 | 1 ( 6.7) |
| Cellulitis | 0 | 0 | 0 |
| Localised infection | 1 ( 0.5) | 0 | 0 |
| Neutropenic sepsis | 1 ( 0.5) | 0 | 0 |
| Pneumonia aspiration | 1 ( 0.5) | 0 | 0 |
| Sepsis | 1 ( 0.5) | 0 | 0 |

**Adverse events leading to discontinuation of anti-cancer medication in first line of treatment (5/6)**

| **System organ class**  **Preferred term (reported in >1  patient)**  **n (%)** | **Pertuzumab +   trastuzumab +  chemotherapy**  **(n=211)** | **Trastuzumab +**  **chemotherapy**  **(n=45)** | **Trastuzumab emtansine-  containing**  **(n=15)** |
| --- | --- | --- | --- |
| **Skin and subcutaneous tissue disorders** | **5 ( 2.4)** | **2 ( 4.4)** | **0** |
| Rash | 3 ( 1.4) | 0 | 0 |
| Pruritus | 2 ( 0.9) | 0 | 0 |
| Erythema | 0 | 1 ( 2.2) | 0 |
| Onychalgia | 1 ( 0.5) | 0 | 0 |
| Pain of skin | 0 | 1 ( 2.2) | 0 |
| Palmar-plantar     erythrodysaesthesia syndrome | 0 | 1 ( 2.2) | 0 |
| **Musculoskeletal and connective tissue disorders** | **2 ( 0.9)** | **0** | **1 ( 6.7)** |
| Arthralgia | 2 ( 0.9) | 0 | 0 |
| Back pain | 0 | 0 | 0 |
| Mobility decreased | 0 | 0 | **1 ( 6.7)** |
| Pain in extremity | 0 | 0 | 0 |

**Adverse events leading to discontinuation of anti-cancer medication in first line of treatment (6/6)**

| **System organ class**  **Preferred term (reported in >1  patient)**  **n (%)** | **Pertuzumab +   trastuzumab +  chemotherapy**  **(n=211)** | **Trastuzumab +**  **chemotherapy**  **(n=45)** | **Trastuzumab emtansine-  containing**  **(n=15)** |
| --- | --- | --- | --- |
| **Blood and lymphatic system disorders** | **1 ( 0.5)** | **0** | **0** |
| Anaemia | 1 ( 0.5) | 0 | 0 |
| **Immune system disorders** | **1 ( 0.5)** | **0** | **0** |
| Anaphylactic reaction | 1 ( 0.5) | 0 | 0 |
| **Psychiatric disorders** | **0** | **1 ( 2.2)** | **0** |
| Agitation | 0 | 1 ( 2.2) | 0 |
